# Language background in early life may be related to neuropsychiatry symptoms in patients with Alzheimer disease

**DOI:** 10.1186/s12877-017-0435-2

**Published:** 2017-02-10

**Authors:** Yi-Chien Liu, Jung-Lung Hsu, Shuu-Jin Wang, Ping-Keung Yip, Kenichi Meguro, Jong-Ling Fuh

**Affiliations:** 1 Neurological Center of Cardinal Tien Hospital, Taipei, Taiwan; 2 Division of Geriatric Behavioral Neurology, CYRIC, Tohoku University, Sendai, Japan; 3 Fu Jen University School of Medicine, Taipei, Taiwan; 4 Department of Neurology, Neurological Institute, Taipei Veterans General Hospital, Taipei, Taiwan; 5 Faculty of Medicine and Brain Research Center, National Yang-Ming University Schools of Medicine, Taipei, Taiwan; 6 Section of Dementia and Cognitive impairment, Department of Neurology, Chang Gung Memorial Hospital, Linkou, 112 Taiwan

**Keywords:** Language background, Dementia, Alzheimer’s disease, Neuropsychiatry symptoms, Language impairment

## Abstract

**Background:**

The relationship between early life experience and the occurrence of neuropsychiatry symptoms (NPSs) in patients with Alzheimer disease (AD) is unclear.

**Methods:**

From 2012 to 2014, we prospectively recruited 250 patients with probable AD from the memory clinic of Taipei Veterans General Hospital. All patients underwent standard assessments, including brain magnetic resonance imaging or computed tomography, neuropsychological tests, neuropsychiatry inventory (NPI-Q) and related blood tests. A linear regression analysis was performed to investigate the relationship between NPSs and age, gender, disease severity, depression, language background (with or without Japanese education).

**Results:**

Among the 250 participants, 113 (45.2%) were women. Their average age was 82.6 years. Of all the participants, 93 (37.2%) had received formal Japanese education, whereas 157 (62.8%) did not receive Japanese education. The participants with Japanese education were slightly younger (83.1 ± 3.6 vs. 81.4 ± 3.4, *P* = 0.006), with a higher proportion of them were women (30.5% vs. 69.8%, *P* < 0.001) and fewer years of total education (10.8 ± 4.5 vs. 7.7 ± 3.2, *P* < 0.001), compared to the participants without Japanese education. NPI-Q scores significantly differed between the two groups (15.8 vs. 24.1, *P* = 0.024). Both disease severity and language background predicted NPI-Q scores.

**Conclusions:**

Language background in early life may be related to NPSs in patients with AD, and this effect is more significant in patients with a lower education level than in those with a higher education level. More NPSs may be the result of negative effects on dominant language or early life experiences.

**Electronic supplementary material:**

The online version of this article (doi:10.1186/s12877-017-0435-2) contains supplementary material, which is available to authorized users.

## Background

Neuropsychiatric symptoms (NPSs), which can be psychotic (delusions and hallucinations), affective (apathy, depressed mood, irritability and anxiety) and, behavioral (euphoria, disinhibition, agitation, aberrant motor activities, sleep disturbance and eating disorder), are the core symptoms of Alzheimer disease (AD) [1]. NPSs is once thought to emerge in people with advanced stage. But it is currently found to manifest in prodromal and all stage of AD. Besides, NPSs is related to rapid cognitive decline, caregiver distress and early institutionalization [2]. In a previous study, the prevalence of NPSs in patients with AD was approximately about 30-40% [3]; the incidence was ranging from 20 to 30% every year [4]. If untreated patients of AD are also considered, the prevalence of NPSs may be as high as 77.8% [5]. Many risk factors for NPSs have been proposed, including biological factors such as age, sex, race, disease severity, and general medical condition. The severity of dementia has been consistently related to NPSs in most studies [3, 6]. However, the findings of studies on these biological risk factors sometimes have been inconsistent or even contradictory [4]. In addition to biological risk factors, studies have emphasized environmental or psychosocial effects on individuals [7–10]. Some studies have described both biological and environmental effects. In a study which recruited 137 elderly Chinese American and 140 Caucasians with and without cognitive impairment from a referral memory clinic found depression was significantly more common in cognitively impaired Chinese Americans compared with cognitively impaired Caucasians. Besides, Chinese Americans were less likely to be on treatment for depression than Caucasians. In that case, depression is not only related to biological factors such as ethnicity but also environmental factors like education and culture [11]. However, few studies have focused on the linkage between early language experience and NPSs in dementia.

In addition to NPSs, AD impairs patients’ cognitive function in multiple domains. Language impairment is one of the earliest and most common symptoms [12]. It often causes communication problems and burdens caregivers [13]. Studies have indicated that language impairment in patients with AD may subsequently lead them to attempt to use languages they used in their childhood or even neologisms [14, 15].

Before World War II (WW II), many Taiwanese people received formal Japanese education in their childhood. Thus, in contrast to their Taiwanese peers who may have received their education after the war or on the mainland, these Taiwanese people can speak Japanese. Moreover, Japanese became their first symbolic language. After the war, the official language of Taiwan was changed to Mandarin Chinese. Therefore, the Japanese speaking ability of Taiwanese people who received formal Japanese education remained at a low fluency level. In everyday life, most members of this group still speak Taiwanese or Mandarin Chinese.

In our previous pilot study, we recruited 21 patients with AD from a memory clinic. We observed that multilingual patients with AD experienced more delusions. Moreover, “language mixing” and “inappropriate emotional response” are believed to be the possible origins of delusions [16]. This study further examined this theory by using a more comprehensive design and including a large sample-size cohort.

## Methods

### Participants

We prospectively recruited 250 patients with AD from the outpatient clinic of Taipei Veterans General Hospital between August 2012 and July 2014. All patients were diagnosed as having AD by a multidisciplinary consensus meeting. The diagnosis of probable AD was made in accordance with the National Institute on Aging-Alzheimer’s Association (NIA-AA) criteria [17]. The disease duration was defined as the period between the initial symptoms reported by a caregiver or a family member and patients’ first visit.

The inclusion criteria included; 1) Patients who were proficient in Mandarin Chinese and were able to complete all our examinations in Chinese; and 2) Patients had to undergo a series of standard assessments, including a detailed clinical dementia history-taking, brain MRI or CT, laboratory tests, and neuropsychological tests. The exclusion criteria included; 1) Patients who were illiterate and aged less than 76 years (those younger than 76 years did not have the chance to receive formal Japanese education); 2) Patients with any possible reversible cause of dementia; and 3) Patients with a history of psychiatric diseases such as schizophrenia.

This study was approved by Ethics Committee of the Taipei Veterans General Hospital, Taipei, Taiwan. Informed consent was obtained from the patients and their family.

### Language status of participants

Most participants in our study who had received Japanese education were capable of using Taiwanese, Japanese and Mandarin Chinese in their daily life. They did not encounter any problems while communicating with other people and understood word meanings in each language. Japanese was the first symbolic language they had learned. Thus, they had continued to watch TV, listen to the radio, and write letters to their friends in Japanese. By contrast, age-matched controls could fluently use only Taiwanese or Mandarin Chinese. They had not received any education in Japanese. Most of them used Mandarin Chinese in everyday life, including in business, government information or letter-writing contexts. In a previous community-based study, we reported a relationship between this complex language environment and dementia prevalence [18].

### MRI analysis and rating scales

Of all participants, 132 (52.8%) underwent whole-brain MRI (GE, 3 T DISCOVERY 750, GE Taiwan) in the clinical assessment. Trans-axial T2 weighted scans, 3D fluid-attenuated inversion recovery images, and high-resolution sagittal T1-weighted images were acquired. The image analysis included a visual rating of medial temporal lobe atrophy (MTA) and posterior cortical atrophy (PA) on T1-weighted images. MTA was rated on a 5-point scale (0 point, absent; 1 point, minimal; 2 points, mild; 3 points, moderate; and 4 points, severe) on the basis of the height of hippocampal formation and the width of the choroid fissure and the temporal horn [19]. PA was rated on a 4-point scale (0 point, absent; 1 point, mild sulcal widening and mild atrophy; 2 points, substantial widening and atrophy; and 3 points, severe atrophy) on the basis of the posterior cingulate and parieto-occipital sulcus and the sulci of the parietal lobes and precuneus [20]. To confirm the consistency of the aforementioned rating methods, several cases were selected and evaluated through a consensus meeting of neurologists.

### Neuropsychological assessment

#### Mini-mental status examination

To evaluate general objective cognitive function, we performed the Mini-Mental Status Examination (MMSE). We used the Mandarin Chinese version of the MMSE which had been translated and validated by one of our authors [21]. The MMSE sub-items were calculated as follows: orientation to time and place (10 points), immediate registration (3 points), attention (5 points), delayed recall (3 points), language (5 points, including naming, repeating phrase, reading and writing), following a three-step command (3 points), and copying a figure (1 point).

#### Clinical dementia rating scale and clinical dementia rating scale Sum of boxes

We evaluated the functional severity of dementia by using the Clinical Dementia Rating (CDR) scale. All clinical information was provided by patients’ caregivers. The CDR Scale Sum of Boxes (CDR-SOB) scores were calculated by adding six domains of functioning scores (memory, orientation, judgement and problem solving, community affairs, home and hobbies, and personal care) [22].

#### Chinese version of the Boston naming test

To assess the language ability of participants, we used the 15-item Mandarin Chinese Version of the Boston Naming Test (C-BNT) [23] during the initial visit.

#### Neuropsychiatric inventory questionnaire

The neuropsychiatric inventory questionnaire (NPI-Q) was administered to examine the frequency and severity of NPSs [24, 25]. All NPI subscales were used in this study, namely delusion, hallucination, agitation, depression, anxiety, euphoria, apathy, disinhibition, irritability, aberrant motor behaviors, sleep disturbances, and eating disturbances. Each subscale score was calculated as the sum of items’ severity (1–3).

#### Geriatric depression scale

We used the 15-item Geriatric Depression Scale (GDS) [26] to evaluate the depression status of our participants.

### Statistical analysis

All analyses were performed using the Statistical Package for Social Sciences, Version 22 (SPSS, Chicago, IL, USA). Demographic variables were compared using Student’s *t* test and the chi-square test when appropriate. A linear regression analysis was performed using NPI-Q scores as the outcome variable and age, sex, CDR-SOB scores, GDS, and language background as predicting variables. To eliminate the possible confounding bias of education and its related effects, we stratified our cases according to whether their education levels were low (<9 years) or high (≥9 years). Nine years of education was the median and mean in our total population. All data used in the analysis are provided in the supporting information.

## Results

### Demographic characteristics of the participants

Of the 250 participants, 113 (45.2%) were women. The average age and the average age and education level of the participants were 82.6 and 9.7 years, respectively. Their disease duration was 48.8 months. Furthermore, 93 (37.2%) participants had received formal Japanese education. The disease severity was similar between the participants with and without Japanese education (Table 1). The MMSE and C-BNT scores did not differ between the two groups (Table 2). Likewise, the disease duration did not differ between them. The participants with Japanese education were slightly younger (83.1 ± 3.6 vs. 81.4 ± 3.4, *P* = 0.006), with a higher proportion of them were women (30.5% vs. 69.8%, *P* < 0.001) and fewer years of total education (10.8 ± 4.5 vs. 7.7 ± 3.2, *P* < 0.001), compared to the participants without Japanese education (Table 1). The GDS scores were similar in the two groups. However, the NPI-Q scores were significantly higher among the participants with Japanese education than among the participants without Japanese education (24.1 ± 33.5 vs. 15.8 ± 23.6, *P* = 0.024).

**Table 1:** Demographic and clinical data of all the study participants and comparison between those with and without Japanese education

	All subjects (*n* = 250)	With Japanese education (*n* = 93)	Without Japanese education (*n* = 157)	*P* value
Age	82.6 ± 3.5	81.4 ± 3.4	83.1 ± 3.6	0.006^*^
Female (*n*, %)	113 (45.2%)	65 (69.8%)	48 (30.5%)	<0.001^*^
Education (years)	9.7 ± 4.3	7.7 ± 3.2	10.8 ± 4.5	<0.001^*^
MMSE	18.9 ± 4.8	18.9 ± 4.7	18.8 ± 4.9	0.964
CDR (*n*, %)				
1	193 (77.2%)	72 (77.5%)	121 (77.1%)	0.473
2	50 (20%)	20 (21.5%)	30 (19.1%)	
3	7 (2.8%)	1 (1.1%)	6 (3.8%)	
CDR-SOB	6.7 ± 3.0	6.6 (2.5)	6.8 (3.2)	0.636
Disease duration (months)	48.8 ± 45.5	45.6 ± 46.5	50.7 ± 44.9	0.390

Note: *MMSE* Mini-Mental State Examination, *CDR* Clinical Dementia Rating Scale, *CDR-SOB* Clinical Dementia Rating Scale Sum of Boxes scores

The *p*-value stand for results of comparison between with/without Japanese education, ^*^ for *p* < 0.05, Values are mean ± SD and number (%)

**Table 2:** Comparison of the results of neuropsychological tests of the study participants with and without Japanese education

	All subjects (*n* = 250)	With Japanese education (*n* = 93)	Without Japanese education (*n* = 157)	*P* value
GDS	3.9 ± 3.3	3.9 ± 3.1	3.9 ± 3.4	0.889
C-BNT	11.6 ± 2.5	11.6 ± 2.5	11.6 ± 2.5	0.948
NPI-Q total	18.9 ± 27.9	24.1 ± 33.5	15.8 ± 23.6	0.024^*^

Note: *GDS* Geriatric Dementia Scale, *C-BNT* Chinese Version of the Boston Naming Test, *NPI-Q* Neuropsychiatric Inventory Questionnaire

The *p*-value stand for results of comparison between with/without Japanese education,^*^ for *p* < 0.05, Values are mean ± SD

### Results of regression analysis

Overall, disease severity (CDR-SOB) and language background both predicted the NPI-Q scores of the participants (*P* = 0.021 and 0.021 respectively; Table 3, Model 1). After stratification was conducted, language background significantly predicted the NPI-Q scores of the low-education group (*P* = 0.014; Table 3, Model 2). In the high-education group, disease severity (CDR-SOB) significantly predicted NPI-Q scores (*P* = 0.012; Table 3, Model 3).

**Table 3:** Linear regression models of NPI-Q scores and Japanese education for all, lower level of education and higher level of education

Predictor	β	Standard error	T	*P* value
Model 1 (All subjects, *n* = 250)				
Age	−0.001	0.496	−0.015	0.988
Gender^a^	−0.004	3.746	−0.064	0.949
GDS	0.039	0.512	0.615	0.539
CDR-SOB	0.148	0.611	2.328	0.021^*^
Japanese education^b^	0.159	3.833	2.320	0.021^*^
Model 2 (Low educational group, *n* = 140)				
Age	0.013	0.725	0.148	0.883
Gender	−0.073	5.438	−0.832	0.407
GDS	0.041	0.785	0.485	0.628
CDR-SOB	0.076	0.956	0.882	0.379
Japanese education	0.220	5.367	2.499	0.014^*^
Model 3 (High educational group, *n* = 110)				
Age	−0.001	0.633	−0.012	0.990
Gender	0.149	5.305	1.365	1.175
GDS	0.013	0.617	0.135	0.892
CDR-SOB	0.246	0.717	2.559	0.012^*^
Japanese education	−0.064	5.653	−0.596	0.552

*MMSE* Mini-Mental State Examination, *CDR-SOB* Clinical Dementia Rating Scale Sum of Boxes scores, *GDS* Geriatric Dementia Scale

Note: Gender^a^: 0 = male, 1 = female, Japanese education^b^: 0 = Without Japanese education, 1 = With Japanese education, β = unstandardized beta coefficient, T = test statistics

^*^ for *p* < 0.05, Subjects received education ≧9 years were classified as high-educational group, <9 years as low-educational group, 9 years of education was the median and mean of total subjects

### NPI-Q and MMSE sub-item analysis

Subsequent analysis indicated a difference in behavioral symptoms mainly in delusion (3.1 vs. 1.9, *P* = 0.043), depression (2.1 vs. 1.2, *P* = 0.033) and anxiety (2.6 vs. 1.3, *P* = 0.004), between the participants with and without Japanese education (Table 4). A further analysis of MMSE sub-items demonstrated that participants with Japanese education scored lower on language-related items (4.0 vs. 4.4, *P* = 0.001; Table 5).

**Table 4:** Comparison of NPI-Q sub-items of the study participants with and without Japanese education

	With Japanese education (*n* = 93)	Without Japanese education (*n* = 157)	*P* value
NPI-Q total	24.1 ± 3.4	15.8 ± 1.8	0.024^*^
Delusion	3.1 ± 4.8	1.9 ± 4.0	0.043^*^
Hallucination	1.0 ± 2.6	1.0 ± 3.1	0.954
Agitation	2.4 ± 4.2	1.6 ± 3.3	0.103
Depression	2.1 ± 4.0	1.2 ± 2.3	0.033^*^
Anxiety	2.6 ± 4.1	1.3 ± 2.9	0.004^*^
Euphoria	0.6 ± 2.3	1.3 ± 2.9	0.213
Apathy	1.9 ± 3.8	1.3 ± 2.8	0.168
Disinhibition	2.1 ± 3.9	1.3 ± 3.1	0.060
Irritability	2.8 ± 4.5	1.9 ± 3.5	0.072
Aberrant behaviors	1.8 ± 3.4	1.2 ± 3.2	0.226
Sleep disturbance	2.0 ± 3.8	1.3 ± 3.0	0.111
Loss of appetite	1.2 ± 2.9	1.0 ± 3.0	0.566

^*^ for *p* < 0.05, Values are mean ± SD

**Table 5:** Comparison of MMSE sub-items of the study participants with and without Japanese education

	With Japanese education (*n* = 93)	Without Japanese education (*n* = 157)	*P* value
MMSE total	18.9 ± 4.7	18.8 ± 4.9	0.964
Orientation	5.1 ± 2.2	4.7 ± 2.4	0.159
Immediate registration	2.8 ± 0.5	2.9 ± 0.3	0.437
Attention	2.8 ± 1.6	2.6 ± 1.9	0.336
Delayed recall	0.8 ± 1.0	0.7 ± 1.0	0.700
Three-step command	1.9 ± 0.9	1.9 ± 1.1	0.875
Language	4.0 ± 0.9	4.4 ± 0.8	0.001^*^
Copying figure	0.6 ± 0.4	0.6 ± 0.4	0.920

^*^ for *p* < 0.05, Values are mean ± SD

### MRI results

The results of the MRI visual rating scale revealed no differences in MTA and PA between the participants with and without Japanese education (MTA scores: 4.2 ± 1.6 vs. 4.0 ± 1.5, *P* = 0.663; PA scores: 2.8 ± 1.2 vs. 2.6 ± 1.0, *P* = 0.195).

## Discussion

The results of this study revealed that the Taiwanese patients with AD who had received Japanese education in childhood might have more NPSs than do those who did not receive Japanese education. The relationship was more significant among the patients with AD who had a low educational level. The difference in NPSs between these two groups was confined to the domains of delusion, depression and anxiety. At the same time, we observed that the patients with AD who had received Japanese education obtained lower language-related MMSE sub-item scores than did their counterparts.

Our study results can be attributed to the effects of “language mixing”, which we have demonstrated in a previous pilot study [16]. Our participants had received formal Japanese education for approximately 6 years in their childhood. Although Japanese was their first symbolic language, they used Taiwanese or Mandarin Chinese in most of their daily life. In other words, they were generally unbalanced multilinguals. When they developed dementia, each language may not have degenerated in parallel [27], and they tended to communicate with other people through mixed language [28]. Subsequently, more misunderstanding and inappropriate emotional responses might have been induced. In a previous study, we illustrated this phenomenon by including several typical cases. In the current study, we found that fluctuation because of communication problems might have resulted in more delusion, depression and anxiety in our patients with AD. The combination of these behavioral symptoms has also been described in dementia patients with impaired language function, and delusion has been associated with early life experience [29].

Our results can also be possibly attributed to chronic stress in this group of people with unique life experiences. After WWII, the official language of Taiwan was changed from Japanese to Mandarin Chinese. Thus, people who had received Japanese education were relatively isolated and had fewer opportunities for jobs and education. Chronic stress has been regarded as a risk factor for AD [30], potentially increasing the incidence or accelerating the appearance of it. A longitudinal study reported that, patients with more self-reported psychological stress in midlife developed AD in late-life in a population-based sample followed for 35 years [31]. In animal studies, inflammation and glucose metabolism were used to explain the underlying mechanism [32]. In our study, more NPSs could also be regarded as an early and a crucial sign of rapid cognitive decline in the future [33].

In this study, the GDS (self-reported), which was conducted during patients’ first visit did not reveal that they were more depressed than another group of people; however, the sub-item analysis of the NPI-Q (completed by their family or caregivers) suggested that the patients were more depressed. A discrepancy was observed between the two scores. This discrepancy may be attributed to the difference between their sense of themselves and sense of their family or caregiver.

The linkage between NPSs and AD is very complex and is not yet fully understood. Many possible mechanisms have been proposed [34]: 1) NPS may reflect a common underlying brain pathology as AD; 2) NPS may share a common risk factor with AD; 3) NPS may be a psychological reaction caused by cognitive decline due to AD; and 4) NPS may synergistically interact with other biological factors and cause rapid decline linked with AD.

## Conclusion

In this study, we found that early life language experience in childhood may be related to more NPSs in dementia in late life. Language mixing and chronic stress may have contributed to the results. However, the relationship between NPSs and AD remain unclear. We may partially answer this question after following our patients for a longer time in future studies.

### Limitations

In this study, we did not examine the socioeconomic status of our participants, which is also a very crucial part of early life experiences. Discrepancy in socioeconomic status might partially explain our results. In our cohort, most patients with Japanese education were women. This was taken into consideration after we included sex as a predictor variable in the regression analysis. However, depression was found to be more prevalent in female patients with dementia previous studies. Thus, the results of our NPI-Q sub-items analysis should be interpreted more carefully. In this study, our subjects are not “pure” monolinguals. Most of them speak mandarin Chinese and Taiwanese even without Japanese education. We may need one more group of “monolingual” AD patients to illustrate the effects of language on NPSs.

## Additional file

Additional file 1: All the statistic results of this study can be calculated from our additional file 1 (XLS 368 kb)
